# Errata

**Published:** 1880-03-20

**Authors:** 


### EDITORIAL AND MISCELLANEOUS.

---

*Errata.*—In the article by Dr A. G. HOBBS, on "Digitalis," in the January number, occur the following errors: On 2d page, 23d line, *fibre* should be *fibres;* and in 5th line from bottom, *systol* should be *systole.* On 4th page, 11th line, *toned* should be *torn;* in 23d line, *veins'* should be *veinous;* and in 6th line from bottom, *imminent* should be *immense.*

On page 476 of our December number, the items accredited to *J. S. Jones* were furnished by T. S. JONES, M.D., of Jackson, Louisiana.

